# Expression of a Cu,Zn superoxide dismutase typical for familial amyotrophic lateral sclerosis increases the vulnerability of neuroblastoma cells to infectious injury

**DOI:** 10.1186/1471-2334-7-131

**Published:** 2007-11-12

**Authors:** Miriam Goos, Wolf-Dieter Zech, Manoj Kumar Jaiswal, Saju Balakrishnan, Sandra Ebert, Timothy Mitchell, Maria Teresa Carrì, Bernhard U Keller, Roland Nau

**Affiliations:** 1 Department of Neurology, Georg-August-University of Göttingen, Göttingen, Germany; 2 Department of Physiology, Georg-August-University of Göttingen, Göttingen, Germany; 3 Dept. of Biology, University of Rome "Tor Vergata", Rome, Italy; 4 Laboratory of Neurochemistry, Fondazione S. Lucia IRCCS, Rome, Italy; 5 Glasgow Biomedical Research Centre, University of Glasgow, Glasgow, UK

## Abstract

**Background:**

Infections can aggravate the course of neurodegenerative diseases including amyotrophic lateral sclerosis (ALS). Mutations in the anti-oxidant enzyme Cu,Zn superoxide dismutase (EC 1.15.1.1, SOD1) are associated with familial ALS. Streptococcus pneumoniae, the most frequent respiratory pathogen, causes damage by the action of the cholesterol-binding virulence factor pneumolysin and by stimulation of the innate immune system, particularly via Toll-like-receptor 2.

**Methods:**

SH-SY5Y neuroblastoma cells transfected with the G93A mutant of SOD1 typical for familial ALS (G93A-SOD1) and SH-SY5Y neuroblastoma cells transfected with wildtype SOD1 were both exposed to pneumolysin and in co-cultures with cultured human macrophages treated with the Toll like receptor 2 agonist N-palmitoyl-S-[2,3-bis(palmitoyloxy)-(2RS)-propyl]-[R]-cysteinyl-[S]-seryl-[S]-lysyl-[S]-lysyl-[S]-lysyl-[S]-lysyl-[S]-lysine × 3 HCl (Pam_3_CSK_4_). Cell viability and apoptotic cell death were compared morphologically and by in-situ tailing. With the help of the WST-1 test, cell viability was quantified, and by measurement of neuron-specific enolase in the culture supernatant neuronal damage in co-cultures was investigated. Intracellular calcium levels were measured by fluorescence analysis using fura-2 AM.

**Results:**

SH-SY5Y neuroblastoma cells transfected with the G93A mutant of SOD1 typical for familial ALS (G93A-SOD1) were more vulnerable to the neurotoxic action of pneumolysin and to the attack of monocytes stimulated by Pam_3_CSK_4_ than SH-SY5Y cells transfected with wild-type human SOD1. The enhanced pneumolysin toxicity in G93A-SOD1 neuronal cells depended on the inability of these cells to cope with an increased calcium influx caused by pores formed by pneumolysin. This inability was caused by an impaired capacity of the mitochondria to remove cytoplasmic calcium. Treatment of G93A-SOD1 SH-SY5Y neuroblastoma cells with the antioxidant N-acetylcysteine reduced the toxicity of pneumolysin.

**Conclusion:**

The particular vulnerability of G93A-SOD1 neuronal cells to hemolysins and inflammation may be partly responsible for the clinical deterioration of ALS patients during infections. These findings link infection and motor neuron disease and suggest early treatment of respiratory infections in ALS patients.

## Background

Infections may increase the risk or accelerate the progression of various neurodegenerative disorders including Alzheimer's disease, Parkinson's disease, stroke and amyotrophic lateral sclerosis (ALS) [1,2]. Moreover, patients with neurodegenerative diseases are more susceptible to systemic, in particular lung infections than healthy persons subsequent to swallowing disturbances and a decreased strength of their respiratory muscles. In support of the link between infections and ALS, an epidemiological study found evidence for infection with Mycoplasma spp. in the blood of more than 80% of patients suffering from ALS and in less than 10% of age-matched control subjects [3].

ALS is an ultimately lethal disease with a high inter-subject variation of progression. It is characterized by the degeneration of cortical and spinal motor neurons. ALS appears to be a multifactorial disease, where motor neuron degradation is initiated by mitochondrial dysfunction or/and by enhanced motor neuron excitability. Mitochondrial function can be disturbed by mutations in the gene encoding Cu,Zn superoxide dismutase (EC 1.15.1.1, SOD1). Clinically, ALS occurs both sporadically and as a familial form. In 5–10% ALS is a familial disease, and approximately 20% of the familial ALS cases are caused by a mutation in the gene encoding SOD1 [4,5]. The point mutation G93A is one of those occurring in familial amyotrophic lateral sclerosis (FALS). Here, in position 93 the amino acid glycine is replaced by alanine in the SOD1 enzyme. Families with the G93A-SOD1 mutation are indistinguishable from sporadic ALS by clinical and pathologic criteria [5]. An in-vitro model to study the cellular alterations associated with mutations of SOD1 was constructed by transfection of the human neuroblastoma cell line SH-SY5Y with G93A-SOD1 [6]. This particular mutation was chosen, because it does not affect the activity of SOD1.

A significant inflammatory component contributes to the pathology of ALS [7,8]. This comprises elevated tissue levels of cyclooxigenase-2 and various cytokines and chemokines in the CNS tissue of ALS patients and mouse models [7,9,10] and the presence of activated microglial cells as demonstrated in post mortem spinal cord tissue of ALS patients [10,11] and by positron emission tomography using [^11^C](R)-PK11195 in living ALS patients [12]. The presence of activated microglial cells in the vicinity of neuronal death in ALS suggests that stimulants of microglial activation are produced by stressed neurons. Conversely, activated microglia can injure neurons both in vitro and in vivo [13-16]. The simultaneous action of host-derived and exogenous stimulants can lead to an additive or supra-additive microglial activation [17]. We hypothesize that in neurodegenerative diseases preactivated microglia are more susceptible to stimulation by infectious agents (e.g., via TLR agonists) and can therefore cause stronger neuronal damage during the course of an infection by the release of nitric oxide, reactive oxygen species and cytokines.

Neuronal injury in infection can originate from systemic inflammation, stimulation of local immunocompetent cells and direct action of bacterial toxins on neurons [1,18]. During CNS infections, Streptococcus pneumoniae primarily causes damage by the direct action of the cholesterol-binding pore-forming hemolysin pneumolysin and through microglia/monocyte activation by agonists of receptors of the innate immune system, particularly Toll-like receptor 2 (TLR2) [18,19]. Both mechanisms may be also of importance in patients with neurodegenerative diseases during extracerebral infections. Here we demonstrate the particular vulnerability of G93A-SOD1 transgenic neuroblastoma cells to both modes of infectious injury.

## Methods

### SH-SY5Y G93A-SOD1 and Wt-SOD1 neuroblastoma cell cultures and measurement of cell viability

Transfected human neuroblastoma cell lines constitutively expressing either wild-type (Wt) human SOD1 or the G93A mutant of this enzyme associated with familial amyotrophic lateral sclerosis (FALS) were previously described [6]. They were routinely maintained in Dulbecco's MEM-F12 (Gibco, Invitrogen, Karlsruhe, Germany) containing 15% fetal calf serum (FCS), 100 U/ml penicillin and 100 μg/ml streptomycin (Invitrogen, Karlsruhe, Germany) at 37°C at a humidified atmosphere with 5% CO_2_. Cell lines were kept in selection by addition of 200 μg/ml geneticin (G418 sulfate, Gibco, Invitrogen, Karlsruhe, Germany); geneticin was removed two days before performing the experiments.

For investigation of the differences in vulnerability to pneumolysin both cell lines were seeded into 96-well plates at a density of 10^5 ^cells/cm^2^. Cultures were treated with medium that contained pneumolysin at a concentration of 0.5 μg/ml. After three hours of exposure cell viability was determined by use of the WST-1 cell proliferation reagent (Roche Applied Science, Mannheim, Germany). The assay is based on the cleavage of the tetrazolium salt WST-1 by active mitochondria, which produces a soluble formazan. Cells were incubated with WST-1 for 2 hours. Then, the formazan dye formed was quantified by measuring the optical density at 490 nm by use of a Genios multiplate reader (Tecan, Crailsheim, Germany). The absorbance directly correlates with the number of metabolically active cells.

### Pneumolysin

Pneumolysin was purified after overexpression of the recombinant toxin in Escherichia coli strain JM109 by hydrophobic and ion exchange chromatography as described before [20]. Toxin purity was assessed by SDS-polyacrylamide gel electrophoresis followed by Coomassie-blue staining which showed a single 52 kD band accounting for 95% of the protein. Endotoxin content of purified pneumolysin was determined using the Limulus amebocyte lysate kinetic-QCL kit (Cambrex, Nottingham, United Kingdom). The purified protein had less than 0.6 endotoxin units per μg of protein, i.e. a very low level which is unlikely to have a biological effect.

### Antioxidant N-acetylcysteine (NAC)

To examine the effect of the antioxidant N-acetylcysteine (NAC) (Sigma, Deisenhofen, Germany) on cell viability, SH-SY5Y G93A-SOD1 and SH-SY5Y Wt-SOD1 human neuroblastoma cells were kept in culture medium containing 1 mM NAC for periods of 24 and 72 hours prior to the experimental procedure. After exposure to pneumolysin for a period of 3 hours cell viability was determined by the WST-1 test.

### Preparation of human macrophages

Human macrophages were derived from peripheral blood mononuclear cells [21]. Shortly, after centrifugation over a Ficoll-Hypaque density gradient, mononuclear cells were plated in RPMI-1640 + 10% FCS and maintained at 37°C in an atmosphere containing 5% CO_2_. Monocytes were allowed to adhere and were then cultivated until differentiation into macrophages for 10 to 14 days as assessed by morphologic criteria such as adherence of the cells and the sprouting of ramifications and functional properties. CD-68 staining showed 98–99% purity of the macrophage cultures.

### SH-SY5Y G93A-SOD1 and Wt-SOD1 neuroblastoma and human macrophage co-culture

For co-culture experiments 5 × 10^4 ^neuroblastoma (G93A-SOD1 or Wt-SOD1) cells/well were seeded on glass coverslips in 24 well plates and allowed to adhere for 24 hours. Trypsin was used initially to separate the neuroblastoma cells, but was washed out twice by centrifugation and PBS washing. The following day after PBS washing and change of culture medium human macrophages were mechanically displaced with a cell scraper and added in concentrations of 10^5 ^cells/well. Co-cultures were maintained for 24 hours in RPMI 1640 medium (Biochrom, Berlin, Germany) containing 10% FCS at 37°C with 5% CO_2 _prior to stimulation experiments. The macrophages did not come into contact with trypsin during this procedure to avoid an activation prior to the experiments.

For activation of macrophages co-cultures were exposed to N-palmitoyl-S-[2,3-bis(palmitoyloxy)-(2RS)-propyl]-[R]-cysteinyl-[S]-seryl-[S]-lysyl-[S]-lysyl-[S]-lysyl-[S]-lysyl-[S]-lysine × 3 HCl (Pam_3_Cys-SKKKK × 3 HCl, EMC Microcollections, Tuebingen, Germany; Pam_3_CSK_4_) [22] in concentrations of 10 μg/ml. After 72 hours neuron-specific enolase was determined in 250 μl of the culture supernatant with a luminescence enzyme immunoassay (LIA) using the Liaison^® ^Analyser from Byk Sangtec and reagents from Diasorin (Dietzenbach, Germany). Cells were fixated with 4% formaldehyde for staining procedures.

### Measurement of intracellular calcium levels

Changes in the cytosolic calcium ([Ca^2+^]_i_) were measured in SH-SY5Y cells expressing either the Wt-SOD1 or the G93A-SOD1 gene attached to glass coverslips after 2–5 days in culture. Cell layers were incubated with RPMI-1640 + 10% FCS (Gibco, Invitrogen, Karlsruhe, Germany) containing 10 μM fura-2 AM at 37°C for 30 min. The RPMI-1640 medium used contains 0.846 mM Ca ^2+ ^(supplier's data). Cells were rinsed with RPMI and further incubated for 20 min at 37°C to allow complete deesterification. Changes in [Ca^2+^]_i _were measured using a CCD camera system (TILL Photonics, Martinsried, Germany) [23,24]. A computer-controlled monochromator (Polychrome II, TILL Photonics) was connected to an Axioscope microscope (Zeiss, Goettingen, Germany) via quartz fiberoptics and a minimum number of optical components for maximum fluorescence excitation (objective Achroplan W 63×, 0.9 W). The CCD camera displayed 12-bit dynamics and an A/D converter with 12.5 MHz sampling rate.

Calcium changes in defined regions of interest (ROIs) were monitored online using the TILL Vision Software V3.3 (TILL Photonics, Martinsried, Germany). Background fluorescence was subtracted from the recorded values. The measured fluorescence ratio [R] at wavelengths 360 and 390 nm was used to calculate the intracellular calcium concentration [Ca^2+^]_i _using the equation of Grynkiewicz et al [25]. The K_d _of fura-2 was experimentally determined as 224 nM [23,26]. Excitation of fura-2 was alternatively done at 360 nm and 380 nm, emitted light was directed to a dichroic mirror with mid-reflection at 425 nm filtered by a band pass filter (505–530 nm). Fluorescence ratio F(360)/F(380) was taken as an estimate of the cytosolic calcium concentration and, accordingly, changes of [Ca^2+^]_i _in fura-2 AM loaded cells are shown. Further analysis was performed off-line with the IGOR software (Wavemetrics, Lake Oswego, OR, USA). Bathing solutions were either RPMI-1640 or (in mM) NaCl 140, KCl 2, CaCl_2 _2.5, MgCl_2 _1, HEPES 10, glucose 40, and bovine serum albumin 0.05% at pH 7.3. For nominally Ca^2+^-free solutions MgCl_2 _was substituted for CaCl_2 _without adding EGTA.

The area under the concentration-versus-time curve (AUC) was calculated with the baseline 0 by the equation: In [Ca^2+^]_i_t = _t1_∫^t2 ^[Ca^2+^]_i_•dt with the help of Origin software, version 7.5 (In = fluorescence intensity under the concentration-versus-time curve, [Ca^2+^]_i _= cytosolic calcium, t = time).

### Hemalum staining, CD68 and activated caspase-3 immunocytochemistry and light green staining

Pneumolysin-stimulated SH-SY5Y cell mono-cultures (Wt-SOD1 and G93A-SOD1) were plated on glass coverslips and were fixated with 4% formaldehyde, dehydrated through graded steps of water/ethanol and histolene and then stained with Meyer's hemalum solution (1: 1 dilution in water).

CD68 and light green staining was used to distinguish between human macrophages and the SH-SY5Y cells in co-culture and to visualize the morphology of the cells; immunostaining for activated caspase-3 was used to detect apoptosis in pneumolysin-treated G93A SOD1 cells: fixated cells were permeabilised with Triton X (0.1% in PBS) for 30 minutes and then incubated with CD68 antibody (clone KP1, dilution 1:50, DAKO, Glostrup, Denmark) or activated caspase-3 antibody [rabbit anti-caspase-3 (cleaved), dilution 1:100, kindly donated by Zytomed Systems, Berlin, Germany] for 90 minutes. Secondary anti-mouse biotinylated antibody or secondary anti-rabbit biotinylated antibody (dilution 1:200, both from Amersham Biosciences, Munich, Germany) were added for 45 minutes. Thereafter, cells were treated with avidin-biotin complex (ABC, Vector, Burlingame, CA) for 30 min, and diaminobenzidine was used for visualisation (5 minutes) resulting in a brown staining of the somata of macrophages (CD68)/apoptotic G93A SOD1 cells (Caspase 3). SH-SY5Y cells were counterstained by light green SF yellowish solution (Chroma-Gesellschaft Schmidt & Co, Stuttgart, Germany) after CD68 immunocytochemistry and with hemalum after caspase-3 immunocytochemistry for 1–2 minutes, rinsed in water, dehydrated and mounted with DePeX (Serva, Heidelberg, Germany).

### In-situ tailing (IST)

In order to assess the quantity of cells which had died by apoptosis, formaldehyde-fixated cells on cover slips were treated with 50 μg/mL proteinase K (Sigma) for 15 min at 37°C in a reaction mixture that contained 10 μL of 5× tailing buffer, 1 μL of digoxigenin DNA labeling mix, 2 μL of cobalt chloride, 12.5 U of terminal transferase, and the amount of distilled water necessary to give a volume of 50 μL. After washing, the cells were incubated with 10% FCS for 15 min at room temperature and then washed again. A solution of alkaline phosphatase labeled anti-digoxigenin antibody in 10% FCS (1:250) was placed on the sections for 60 min at 37°C. The color reaction (black) was developed with 4-nitroblue-tetrazolium (NBT) chloride/5-bromine-4-chloride-3-indolyl-phosphate. The cover slips were counterstained with nuclear fast red aluminum hydroxide (reagents from Roche).

### Statistics

Graph Pad Prism Software (GraphPad Software, San Diego, California, USA) was used to perform statistical analyses and graphical presentation. Experiments were reproduced at least three times. Data were expressed as means ± SDs. Groups were compared by two-tailed parametric one-way ANOVA, and p values were adjusted for repeated testing by Bonferroni's multiple comparison test. P < 0.05 was considered to be statistically significant.

## Results

### Increased toxicity of pneumolysin for neuroblastoma cells transfected with G93A-SOD1

**Figure 1:**
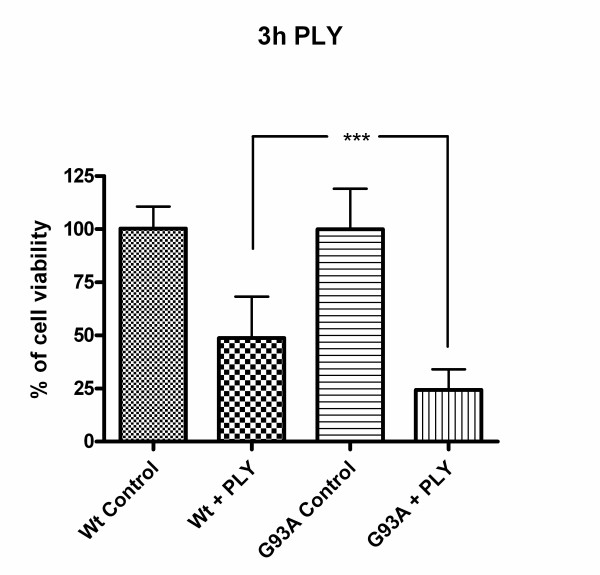
**Toxicity of pneumolysin for Wt-SOD1 and G93A-SOD1 neuroblastoma cells**. Cell viability measured by the WST-1 test after 3 h of incubation with pneumolysin (PLY) at a concentration of 0.5 μg/ml. Values are given in % of mitochondrial metabolic activity of unstimulated control cells ± standard deviation (SD). G93A-SOD1 mutant SH-SY5Y cells were more vulnerable to the action of PLY than Wt-SOD1 SH-SY5Y cells (p < 0.0001).

After incubation of Wt-SOD1 and G93A-SOD1 neuroblastoma cells with the pneumococcal virulence factor pneumolysin for 3 hours, the G93A-SOD1 mutant cells showed a significantly decreased cell viability as evidenced by the WST-1 test (24.3 ± 9.6% of the living untreated cells) compared to the Wt-SOD1 cells (48.8 ± 19.5%; p < 0.0001, Fig. 1).

**Figure 2:**
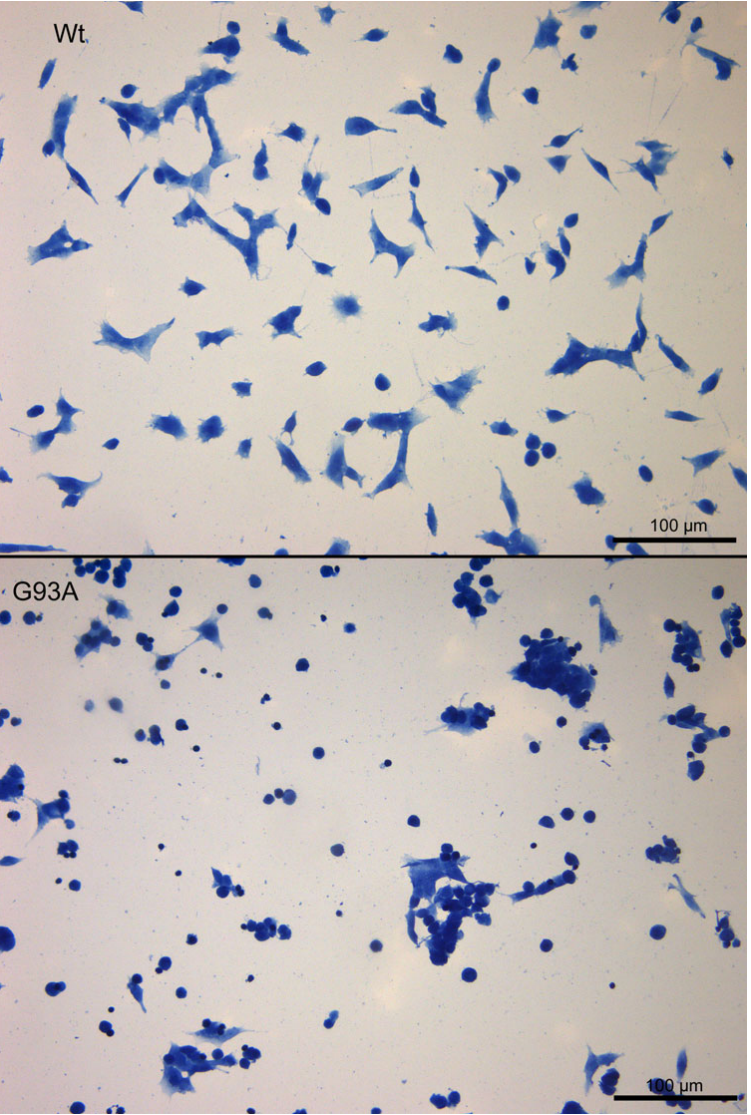
**Toxicity of pneumolysin for Wt-SOD1 and G93A-SOD1 neuroblastoma cells**. Hemalum staining showed a substantially higher density of living Wt-SOD1 SH-SY5Y cells than G93A-SOD1 SH-SY5Y cells after PLY treatment. Please note the shrinkage and clustering of severely damaged/dead G93A-SOD1 neuroblastoma cells.

**Figure 3:**
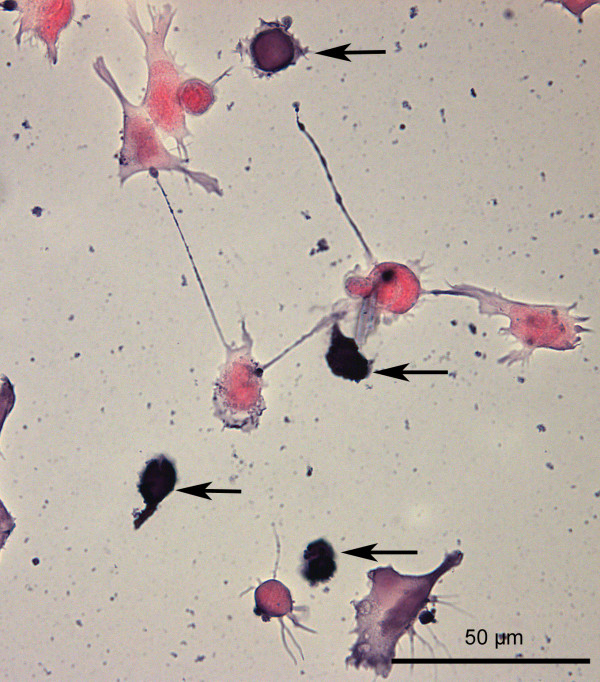
**Toxicity of pneumolysin for G93A-SOD1 neuroblastoma cells**. Apoptotic G93A-SOD1 SH-SY5Y cells after incubation with 0.5 μg/ml PLY for three hours (in-situ tailing, apoptotic cells marked by arrows).

**Figure 4:**
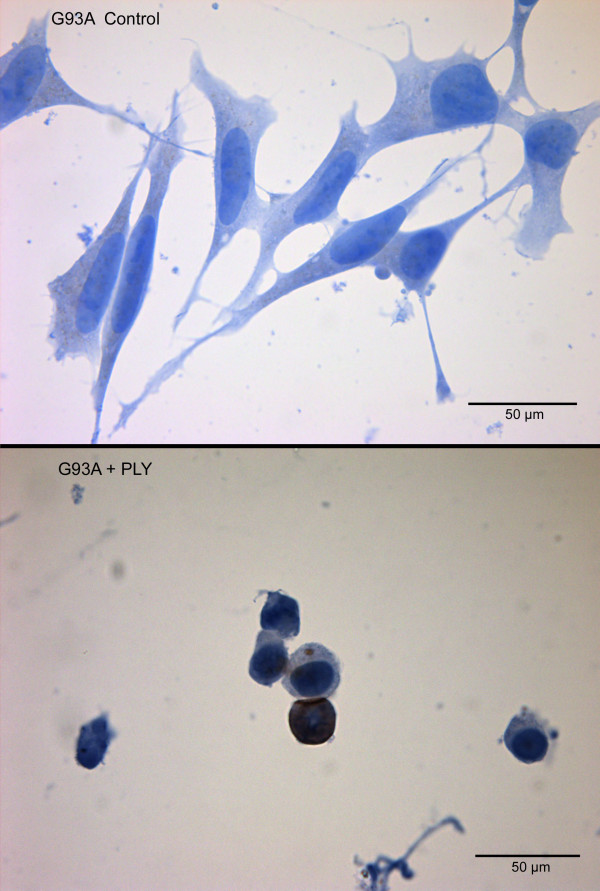
**Toxicity of pneumolysin for G93A-SOD1 neuroblastoma cells**. Apoptotic G93A-SOD1 SH-SY5Y cells after incubation with 0.5 μg/ml PLY for three hours detected by immunocytochemistry for activated caspase-3.

**Figure 5:**
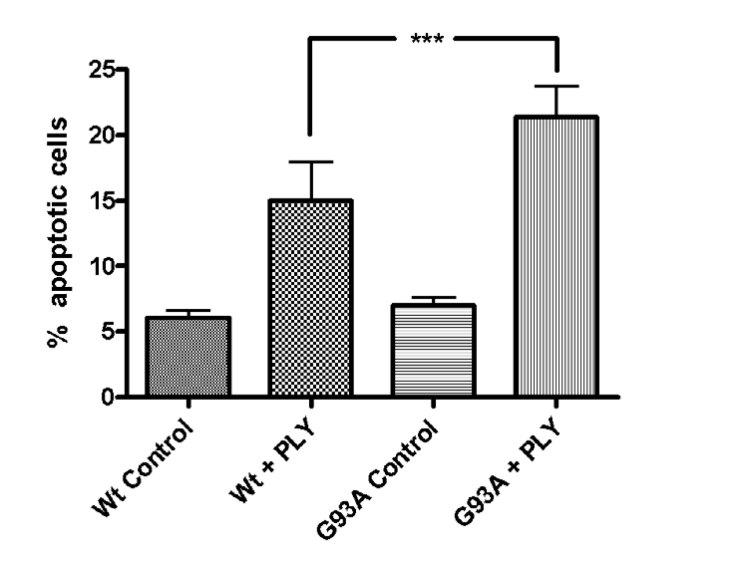
**Toxicity of pneumolysin for Wt-SOD1 and G93A-SOD1 neuroblastoma cells**. Rate of apoptotic cells in Wt-SOD1 SH-SY5Y and G93A-SOD1 SH-SY5Y neuroblastoma cells after incubation with 0.5 μg/ml PLY for three hours (all cells = 100%). Please note the strong difference of the rate of apoptosis in wild-type and G93A-SOD1 transgenic cells exposed to pneumolysin (p < 0.001).

This phenomenon was morphologically confirmed by staining of the cell somata with hemalum. A significantly higher density of living cells after pneumolysin treatment was observed in cultures of Wt-SOD1 cells compared to G93A-SOD1 neuroblastoma cultures (Fig. 2). By in-situ tailing, morphology (arrows) (Fig. 3), and by immunocytochemistry for activated caspase-3 (Fig. 4) of G93A-SOD1 neuroblastoma cells it became apparent that a large proportion of these cells died by apoptosis. The rate of apoptotic neuroblastoma cells in the different treatment groups is presented in Fig. 5.

### Impaired ability of neuroblastoma cells transfected with G93A-SOD1 to cope with the pneumolysin-induced calcium influx

**Figure 6:**
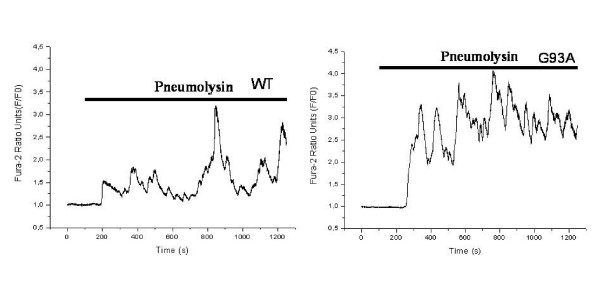
**Strongly elevated calcium influx into G 93A-SOD1 neuroblastoma cells in comparison to wild-type SOD1 cells**. Representative recordings of intracellular calcium concentrations in single cells as measured by the fura-2 AM method.

**Figure 7:**
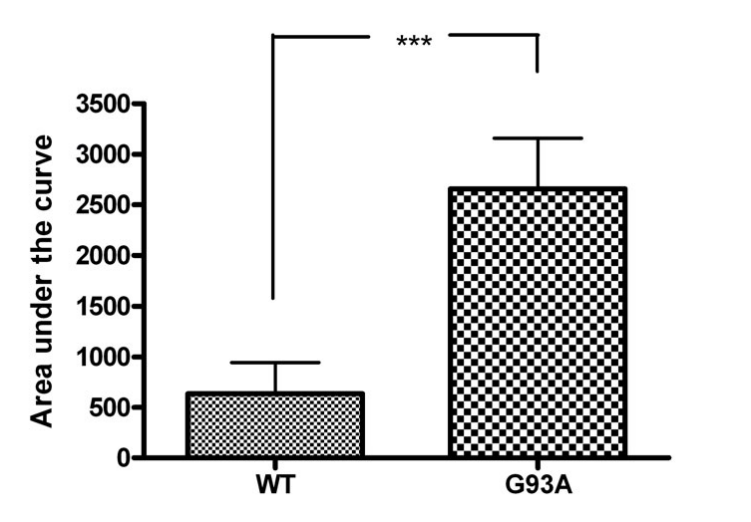
**Strongly elevated calcium influx into G 93A-SOD1 neuroblastoma cells in comparison to wild-type SOD1 cells**. Comparison of the intracellular calcium concentration-versus-time curves (n = 25 cells each; means ± SD; p < 0.0001).

**Figure 8:**
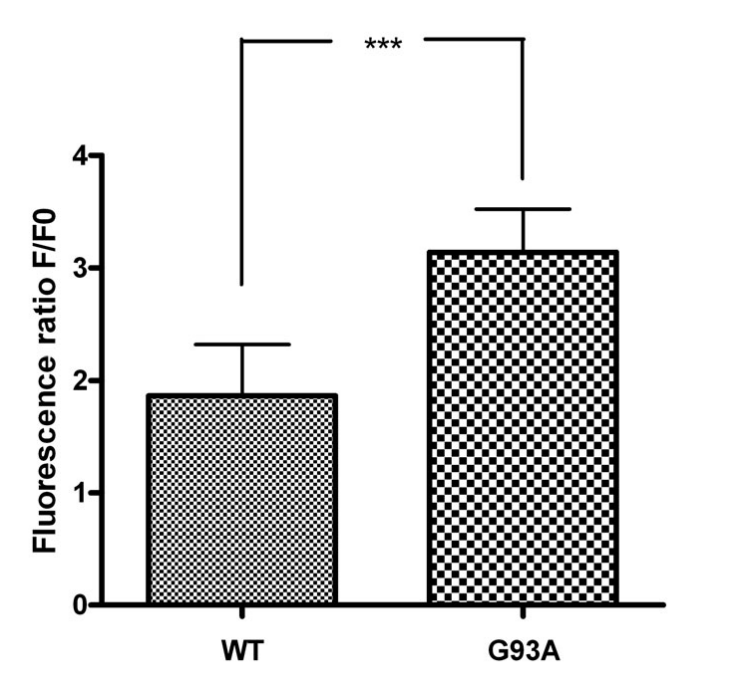
**Strongly elevated calcium influx into G 93A-SOD1 neuroblastoma cells in comparison to wild-type SOD1 cells**. Comparison of the pneumolysin-induced peak calcium intracellular concentrations (n = 25 cells each, means ± SD, p < 0.0001).

For a period of 20 minutes both cell lines were treated with pneumolysin in concentrations of 0.5 μg/ml. During this time cytoplasmic calcium levels were measured in both cell lines. Fig. 6 shows intracellular calcium levels [Ca^2+^]_i _as cytoplasmic calcium-versus-time curves in two representative cells during pneumolysin treatment. The ability of G93A-transfected SH-SY5Y cells to maintain low cytoplasmic calcium levels was strongly reduced. Analysis of the cytoplasmic calcium-versus-time curves of 25 G93A-SOD1 transfected and 25 Wt-SOD1 transfected cells after exposure to pneumolysin for 20 minutes revealed an approximately 4-fold increase of the area under the curve (AUC) in G93A-SOD1 SH-SY5Y cells (634.0 ± 309.0 in Wt-SOD SH-SY5Y versus 2658.0 ± 502.3 in G93A-SOD1 SH-SY5Y cells, p < 0.0001) (Fig. 7). The pneumolysin-induced [Ca^2+^]_i _peak values in G93A-SOD1 cells amounted to approximately 1.6-fold of the corresponding [Ca^2+^]_i _peak values in Wt-SOD1 cells (3.1 ± 0.4 versus 1.9 ± 0.5, p < 0.0001) (Fig. 8).

### The pneumolysin-induced neuronal injury was attenuated by the anti-oxidant N-acetyl-cysteine (NAC)

**Figure 9:**
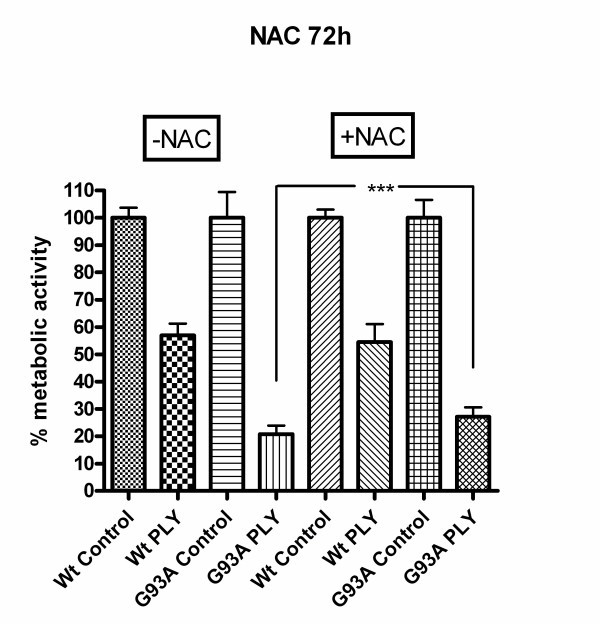
**Radical scavenging by N-acetyl-cysteine**. The pneumolysin-induced neuronal injury in G93A-SOD1 neuroblastoma cells, but not in Wt-SOD1 cells, was attenuated by pre-incubation for 72 hours with the anti-oxidant N-acetylcysteine (NAC) in a concentration of 1 mM (p < 0.001).

**Figure 10:**
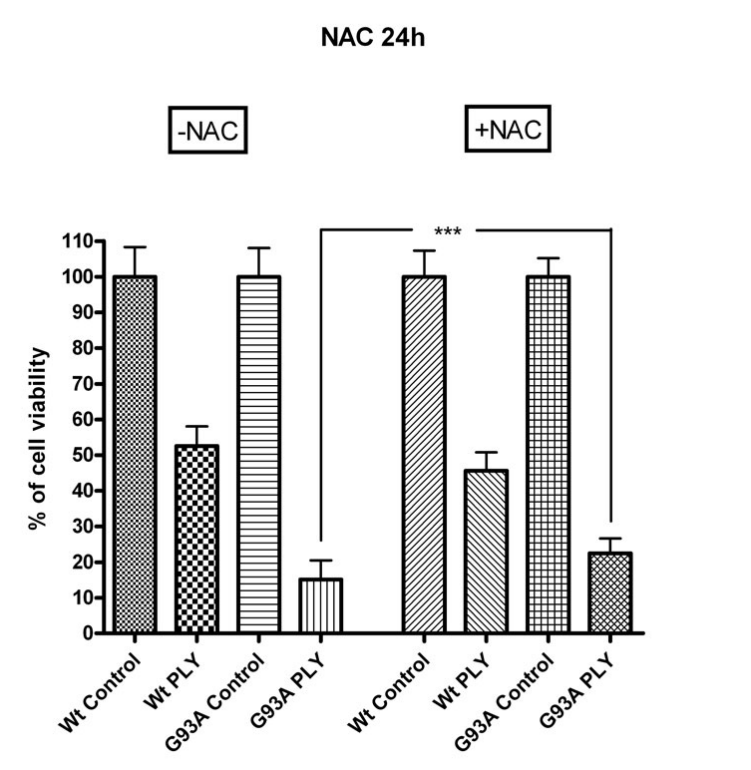
**Radical scavenging by N-acetyl-cysteine**. 24 hours of pre-incubation with the anti-oxidant N-acetylcysteine (NAC) in a concentration of 1 mM also resulted in an attenuation of the pneumolysin-induced neuronal injury in G93A-SOD1 (p < 0.0001), but not in Wt-SOD1 neuroblastoma cells.

Incubation with the antioxidant N-acetyl-cysteine (NAC) in a concentration of 1 mM starting 72 and 24 hours in the absence of pneumolysin suggested a slightly higher mitochondrial metabolic activity of G93A-SOD1 cells compared to G93A-SOD1 cells kept in medium without NAC (difference not significant). After 72 hours of NAC pre-treatment, pneumolysin exposure to G93A-SOD1 cells resulted in a metabolic activity of 27.2 ± 3.5% of G93A-SOD1 control cells not exposed to pneumolysin compared to a metabolic activity of 20.8 ± 3.1% of respective pneumolysin-challenged G93A-SOD1 cells in the absence of NAC (p < 0.001) (Fig. 9). After pre-incubation of G93A-SOD1 cells in NAC-containing medium for a period of 24 hours similar results were observed: NAC exerted a neuroprotective effect on G93A-SOD1 cells treated with pneumolysin as determined by the WST-1 test after 3 hours of pneumolysin exposure (22.5 ± 4.1% mitochondrial metabolic activity of control cells in G93A cells with NAC treatment versus 15.1 ± 5.4% without NAC treatment, p < 0.0001) (Fig. 10).

Contrarily, there was no significant difference in the metabolic activity of NAC-treated and untreated Wt-SOD1 cells after pneumolysin incubation, i.e., NAC did not protect Wt-SOD1 neuroblastoma cells from the toxic action of pneumolysin (Fig. 9 &10) (56.9 ± 4.3% mitochondrial metabolic activity in pneumolysin-treated Wt-SOD1 cells versus 54.5 ± 6.6% in pneumolysin-exposed Wt-SOD1 cells with 72 hours pre-incubation with NAC, p > 0.05).

### Increased vulnerability of neuroblastoma cells transfected with G93A-SOD1 to the attack of monocytes stimulated with the Toll-like receptor-2 agonist Pam_3_CSK_4_

**Figure 11:**
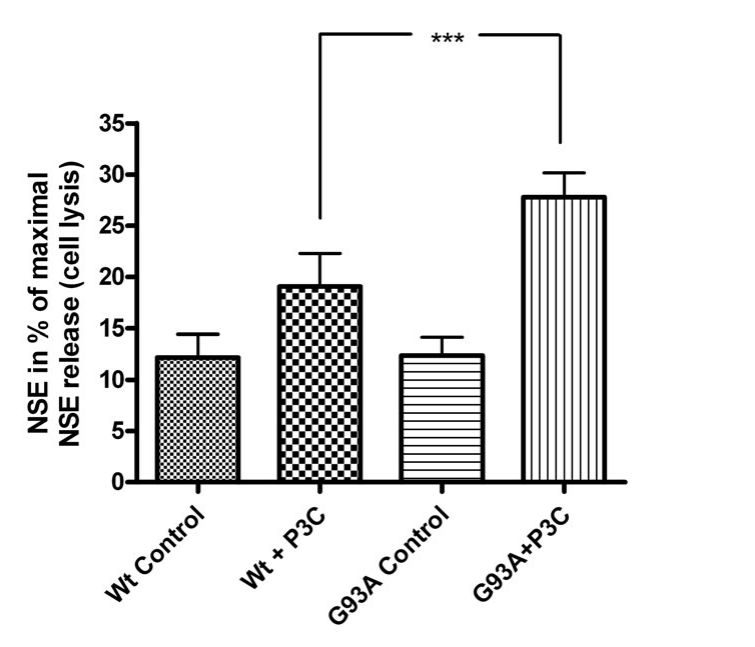
**Vulnerability of G93A-SOD1 and wild-type SOD1 neuroblastoma cells to the attack of monocytes stimulated with Pam_3_CSK_4_**. Release of neuron-specific enolase (values expressed in per cent ± SD of the NSE release induced by cell lysis). After stimulation of monocytes with the Toll-like receptor 2 agonist Pam_3_CSK_4 _G93A-SOD1 SH-SY5Y cells were more severely injured by activated macrophages than Wt-SOD1 cells.

**Figure 12:**
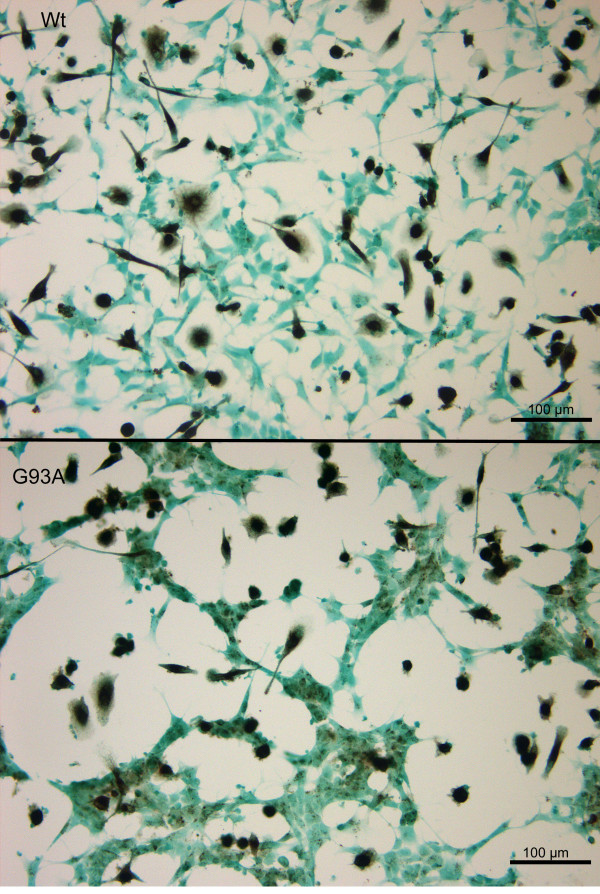
**Vulnerability of G93A-SOD1 and wild-type SOD1 neuroblastoma cells to the attack of monocytes stimulated with Pam_3_CSK_4_**. After staining of Pam_3_CSK_4_-stimulated co-cultures with light green and macrophage staining with CD 68 less neuronal cell somata of G93A-SOD1 SH-SY5Y (lower panel) cells than of Wt-SOD1 cells in equally treated co-cultures (upper panel) were visible. Please note the clustering of severely damaged/dead G93A-SOD1 cells and the area devoid of neuronal cells in the vicinity of groups of macrophages.

**Figure 13:**
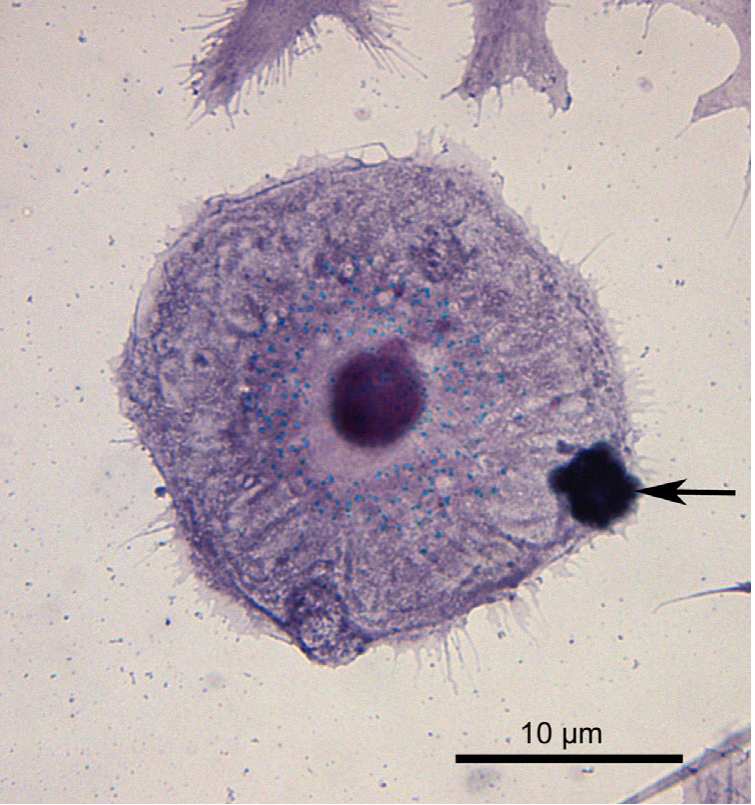
**Vulnerability of G93A-SOD1 neuroblastoma cells to the attack of monocytes stimulated with Pam_3_CSK_4_**. In-situ tailing shows a macrophage internalising an apoptotic nucleus of a G93A-SOD1 neuroblastoma cell (arrow).

After stimulation of human neuroblastoma and macrophage co-cultures with the TLR2 agonist Pam_3_CSK_4 _for a period of 72 hours the release of neuron-specific enolase (NSE) was measured in the culture supernatants and expressed as per cent of the NSE release induced by cell lysis. Co-cultures of macrophages with G93A-SOD1 SH-SY5Y cells showed a significantly higher release of NSE compared to co-cultures with Wt-SOD1 SH-SY5Y cells after stimulation with Pam_3_CSK_4 _(27.8 ± 2.4% vs. 19.0 ± 3.3%; p < 0.001) (Fig. 11). After cell staining with light green and macrophage staining with CD 68 significantly less neuronal cell somata of G93A-SOD1 SH-SY5Ycells per mm^2^ than of equally treated Wt-SOD1 cells were counted by microscopy (Fig. 12). In-situ tailing detected macrophages attacking the apoptotic neuroblastoma cells (arrow) (Fig. 13).

## Discussion

Patients with neurodegenerative diseases can experience irreversible deterioration during infections. In an *in-vitro *model of amyotrophic lateral sclerosis we showed that SH-SY5Y neuroblastoma cells transfected with the G93A mutant of SOD1 typical for familial ALS (G93A-SOD1) are more vulnerable to infectious stimuli than neuroblastoma cells overexpressing normal SOD1. The increased vulnerability was observed after exposure to the bacterial hemolysin pneumolysin and after co-incubation with activated monocytes.

Hemolysins are important virulence factors of a variety of bacteria. The cholesterol-binding hemolysin pneumolysin binds to eukariotic lipid membranes. There it oligomerizes into ring-shaped structures and forms non-selective pores within the lipid bilayer with a diameter of 25–50 nm [27-32]. At sublytic concentrations, pneumolysin rapidly activates Rho and Rac GTPases and leads to the formation of actin stress fibers, filopodia, and lamellipodia. At these low concentrations, pneumolysin does not appear to form macropores, but micropores with ion channel properties [29]. Formation of pores leads to a Ca^2+ ^flux from the extracellular to the intracellular space causing an increase of intracellular Ca^2+ ^in the micromolar range and affecting cell survival. The increase of the intracellular Ca^2+ ^concentration originates from the influx of extracellular Ca^2+ ^and not from mobilization of intracellular stores and is mediated by the pneumolysin pore itself and not via voltage-gated Ca^2+ ^channels [30]. The massive Ca^2+ ^influx causes activation of p38 MAPK, opening of the mitochondrial permeability transition (MPT) pore and consecutive caspase activation. At low concentrations, pneumolysin primarily leads to apoptosis [30]. The amount of pneumolysin released by S. pneumoniae can be influenced by the onset and choice of antibiotic therapy [33].

SH-SY5Y cells transfected with G93A-SOD1 suffer from a decreased mitochondrial membrane potential and an elevated intracellular Ca^2+ ^concentration already at rest as determined by fluo-3 AM staining [6]. We also found a slight elevation of the intracellular Ca^2+ ^already in unchallenged G93A-SOD1-transfected cells. After exposure to pneumolysin, the capacity of G93A-SOD1 cells to control cytosolic Ca^2+ ^by transport to the extracellular space or to intracellular storage sites was much lower than the capacity of neuroblastoma cells not transfected with mutant SOD1. This accounted for the increased vulnerability of SH-SY5Y cells possessing a mutant G93A-SOD1 gene to low concentrations of pneumolysin.

The anti-oxidant N-acetylcysteine has been shown to restore mitochondrial function and to lower the production of reactive oxygen species in neuroblastoma cells expressing mutant SOD1 [34]. In this study, it inhibited pneumolysin-induced neurotoxicity. Accordingly, in G93A-SOD1 transgenic mice, a 1% solution of N-acetylcysteine administered as drinking water from 4–5 weeks of age delayed the onset of motor dysfunction and prolonged survival [35]. The in-vitro and in-vivo data available suggest that N-acetylcysteine, a drug with low toxicity used to liquefy the pulmonary secretion in pneumonia and to protect the liver after paracetamol poisoning, should be explored in a randomized trial concerning its ability to lower the speed of progression of amyotrophic lateral sclerosis in humans.

Within the CNS, microglial cells and meningeal and perivascular macrophages participate in the resistance to infection, removal of cell debris from sites of injury and promotion of tissue repair [36-38]. Microglia and monocytes/macrophages are derived from the same progenitor cells, express TLRs and other receptors mediating innate immunity [39-42]. Because primary human microglia are not readily available and tumor-derived microglial cell lines behave differently from primary microglia upon stimulation [43], in this study we used primary cultures of monocytes/macrophages from the systemic circulation of blood donors.

Monocytes and microglia activated by single TLR agonists can kill neurons [[13-16,44,45], present data]. Mitochondrial damage contributes to neuronal death both in inflammation and ALS [46,47]. Neurotoxicity of microglia has been observed in vitro following stimulation with the TLR4 agonist lipopolysaccharide (LPS) [44,45]. and analogues of bacterial DNA (TLR9 agonist) [13]. The neurotoxic mechanisms of microglia and macrophages involve generation of nitric oxide (NO) and other reactive oxygen species [13,44]. Here we demonstrate that activation of macrophages by the TLR2 agonist Pam_3_CSK_4 _can also cause neuronal death, and that neuroblastoma cells expressing G93A-SOD1 are more susceptible to the attack of activated immune cells than those expressing wild-type SOD1.

In the brain of healthy individuals, microglia are in a resting state. Conversely, in several neurodegenerative diseases, endogenous compounds present in the extracellular space lead to a chronic activation of microglia [1]. Activated microglia are observed in various diseases including autoimmune- and infection-mediated inflammation, trauma, ischemia and neurodegeneration [37,38]. In inherited ALS, after an initial phase of the disease predominated by motor neuron damage caused by mutant SOD, in a later phase of disease progression is linked to the inflammatory response of microglia [8].

Low concentrations of different TLR agonists can cause additive or supra-additive stimulation rendering microglial cells very susceptible to bacterial products at low concentrations [48,49]. Co-stimulation of microglia with host-derived compounds (β-amyloid, fibronectin, advanced glycation end products) and bacterial products can lead to an additive or supra-additive microglial activation [17,50-52]. In addition to their greater vulnerability to bacterial hemolysins, increased susceptibility of neurons expressing the G93A mutant in their SOD1 to the attack of activated immune cells may be the pathophysiological basis of the vulnerability of the nervous system of patients with motor neuron disease to systemic infections. Endogenous compounds deposited in the extracellular space (e.g., β-amyloid) [17], entering the brain through the leaky blood-brain barrier (e.g., fibronectin) [51] or released by dying neurons and oligodendrocytes (advanced glycation end products) [50,52] can transform microglia from a dormant into an activated state, which renders them more susceptible to the stimulation by bacterial products.

## Conclusion

Human immune cells of the monocyte/macrophage/microglia type activated by a TLR2 agonist can kill neurons. Neuronal cells expressing a SOD1 mutant frequently encountered in familial cases of amyotrophic lateral sclerosis are more vulnerable to the direct action of the bacterial hemolysin pneumolysin and to the attack of activated immune cells than neuronal cells expressing wild-type SOD1. In many neurodegenerative diseases, microglia are chronically stimulated by host-derived compounds and change their phenotype from a dormant to the activated state. Activated microglia are more susceptible to stimulation with bacterial products than resting microglia. Increased vulnerability of neurons and increased susceptibility of immune cells to bacterial products probably are two causes why patients with neurodegenerative diseases frequently deteriorate during infections. Our in vitro findings must be confirmed in animal experiments and human studies, before conclusions concerning changes in treatment can be drawn. A therapeutic strategy directed at minimizing the release of proinflammatory/toxic bacterial compounds like TLR-agonists and pneumolysin can be achieved either by the early treatment of infections with antibiotics and/or by the use of bactericidal drugs which minimize the release of bacterial products through inhibition of bacterial protein synthesis [53-55]. Both strategies may be beneficial in patients suffering from neurodegenerative diseases.

## Competing interests

The author(s) declare that they have no competing interests.

## Authors' contributions

MG, WDZ and SE carried out the SH-SY5Y cell culture experiments. They established the primary cultured human macrophage preparation and the co-cultures of SH-SH5Y and human macrophages. They developed methods to quantify neuronal damage via WST-1 test, NSE measurement, in-situ tailing and staining methods of the cells and performed the statistical analysis of the stimulation experiments. MKJ, SB and BUK carried out the measurement of pneumolysin-induced calcium influx in the cells. TM provided endotoxin-free pneumolysin used in the experiments. Transfected human neuroblastoma cell lines expressing either wild-type (Wt) human SOD1 or the G93A mutant of this enzyme associated with familial amyotrophic lateral sclerosis (FALS) were produced by MTC. MTC and TM discussed the results in the context of previous work and helped in preparing the manuscript. RN proposed the project, participated in the design of the experiments, supervised the laboratory work and helped to draft the manuscript. All authors read and approved the final manuscript.

## Pre-publication history

The pre-publication history for this paper can be accessed here:


